# A complex variant t(3;15) (q26;q13) representing cryptic/masked acute promyelocytic leukaemia with a novel breakpoint of chromosome 15—a case report

**DOI:** 10.3332/ecancer.2013.340

**Published:** 2013-08-15

**Authors:** Sandhya Appachu, Chintaparthi Obulareddy, Nagesh T Sirsath, Kuntejowdahalli C Lakshmaiah, Prasanna Kumari

**Affiliations:** 1 Department of Medical Oncology, Kidwai Memorial Institute of Oncology, Banglore, Karnataka 560029, India; 2 Department of Pathology, Cytogenetics Unit, Kidwai Memorial Institute of Oncology, Banglore, Karnataka 560029, India

**Keywords:** APML, variant translocation, ATRA

## Abstract

Acute promyelocytic leukaemia (APML) is a biologically and clinically distinct variant of AML, currently classified as acute myeloid leukaemia with recurrent cytogenetic anomalies t(15;17) (q22;q21), promyelocytic leukaemia-retinoic acid receptor alpha, diagnosis regardless of blast count in the World Health Organization classification system. It is one of the curable malignancies, has a unique clinical presentation, often with disseminated intravascular coagulation, and has a targeted therapy for its treatment in the form of all trans retinoic acid (ATRA) and arsenic trioxide (ATO). Here, we report a complex type of variant APML t(3;15) (q26;q13), the need for conventional karyotyping for diagnosing such rare variants, and its response to ATRA and ATO.

## Introduction

Acute promyelocytic leukaemia (APML) constitutes 5–10% of acute myeloid leukaemia (AML) and is characterised by a reciprocal translocation between chromosomes 15 and 17 [1], leading to the fusion of retinoic acid receptor alpha (RARA) gene on chromosome 17 at q21 and promyelocytic leukaemia (PML) gene on chromosome 15 at q22. However, t(15;17) is absent in around 8% of patients diagnosed with APML [2]. Cases lacking t(15;17) are divided as cryptic and complex APML and AML with a variant RARA translocation [2, 3]. In cryptic and complex APML, the classic t(15;17) is absent on routine cytogenetic studies, but PML-RARA is present on molecular studies. AML patients with variant RARA translocations are rare.

These include the t(11;17)(q23;q21), t(5;17)(q35;q21), t(11;17)(q13;q21), and der(17) that create STAT5b-RARA fusion. Thus, several cases of variant translocations involving 17q but not 15q have been reported, leading to the suggestion that the break in 17q rather than the one in 15q is the crucial change in the regular t(15; 17). Only one case of APML with t(3; 15) (q21; q22) has been reported in the past, showing involvement of 15q without involvement of 17q [4].

We report an interesting unique case of APML with t(3;15) (q26;q13) on conventional cytogenetics and molecular assay showing PML-RARA fusion, indicating break in chromosome 15q13 in addition to classic PML-RARA fusion.

## Case report

**Figure 1:**
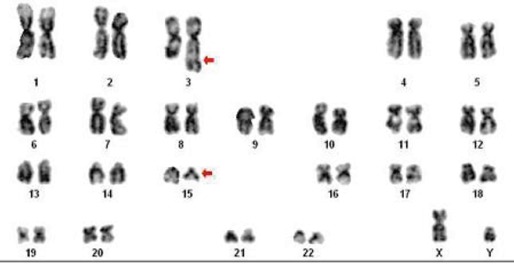
Karyotype: 46, XY, t(3;15) (q26;q13). Arrow indicates breakpoint.

A 15-year-old male presented with complaints of generalised weakness, fever, and gum bleeding for 15 days prior to examination. On examination, he was pale and had digital clubbing, and there was no lymphadenopathy. Cardiac auscultation revealed loud S1, fixed splitting of S2, and ejection systolic murmur in the pulmonary area. The rest of the systemic examination results were within normal limits. At presentation, a haemogram showed Hb 4 g/dL, total count of 1.3 × 109/L, and platelets 15 × 10^9^/L. A peripheral smear showed abundant promyelocytes. Bone marrow aspiration showed hypercellular marrow with 90% promyelocytes with dense granulation and nuclear bilobation; occasional cell showed multiple Auer rods. A cytogenetic study was carried out on cells from bone marrow aspiration. Short-term cultures of 24 and 48 h were set up in RPMI 1640 (GIBCO-BRL) medium supplemented with 20% foetal bovine serum. After 16–18 h, 50 μL of colcemid at a final concentration of 10 μg/mL was added for 30 min followed by hypotonic treatment in 0.075 M KCl, fixation in Carnoy’s fixative. Giemsa (GTG) banding was performed according to the standard protocol. Fifteen metaphases were analysed, which consistently showed 46, XY t(3;15) (q26;q13)karyotype (Figure 1). Reverse transcription-polymerase chain reaction (RT-PCR) revealed PML-RARA fusion.

His chest x-ray showed a dilated right atrium and bilateral hilar congestion. Two-dimensional ECHO was suggestive of congenital heart disease [ostium secondum atrial septal defect (ASD)], dilated right atrium and right ventricle, mild tricuspid regurgitation, and grade I pulmonary hypertension. He had normal left ventricular ejection fraction (EF 60%) and minimal pericardial effusion. The patient was diagnosed as AML M3, intermediate risk with congenital heart disease-ostium secondum ASD. Cardiac consultation was taken, and the patient was planned for corrective ASD closure at a later date. In view of his underlying cardiac disease, anthracyclins were not considered for induction. His corrected QTc was 0.47 s, and he was planned for an induction regimen with all trans retinoic acid (ATRA)— 45 mg/m^2^in two divided doses and arsenic trioxide (ATO)—0.15 mg/kg/day. He had an eventful induction with differentiation syndrome on day 4 from which he recovered with steroids and a right lower lobe pneumonia toward mid-induction, which resolved with parenteral antibiotics. He attained haematological remission on day 45 of successful induction.

## Discussion

Around 8% of patients diagnosed with APML will lack classic t(15;17) [2]. These cases are either cryptic or complex APML that share the same phenotype, prognosis, and sensitivity to ATRA and ATO as classic APML or ‘AML with a variant RARA translocation’ [3]. Cryptic and complex APML is characterised by the absence of the classic t(15;17) on routine cytogenetic studies, but PML-RARA is present on molecular studies. The European working party characterised the rare APML cases lacking the classic t(15;17) on routine cytogenetic studies [3]. In their analysis, cryptic or masked APML with submicroscopic insertion of RARA into PML leading to the expression of the PML-RARA transcript accounted for 4% cases, while 2% had complex variant translocations involving chromosomes 15, 17, and an additional chromosome. These complex variant translocations were sub-classified as (a) complex variant t(15;17) due to a three-way balanced translocation involving 15q22, 17q21, and another chromosome; (b) simple variant t(15;17) involving 15q22 or 17q21 with another chromosome; and (c) a very complex case [3]. Our patient had t(3;15) (q26;q13) positive for PML-RARA representing a case of complex variant APML with a novel breakpoint of chromosome 15.

The term ‘AML with a variant RARA translocation’ is used by the World Health Organization (WHO) to designate a subset of AMLs morphologically similar to APL, but lacking both t(15;17) by cytogenetics and PML-RARA by FISH and RT-PCR. Compared with classic APML, these leukaemias often exhibit significant differences in malignant phenotype and sensitivity to ATRA. They show different variant translocations involving RARA and other partner genes (Table 1). Variant translocations involving 17q21 but not 15q22 have been reported, leading to the suggestion that the break in 17q21 rather than 15q22 is the crucial change in the regular t(15;17). Only one case of APML with t(3;15) (q21;q22) has been reported, showing involvement of 15q without involvement of 17q21 [4]. The present case was unique by non-involvement of 15q22 and 17q21 breakpoints. Further it may be a cryptic or masked APML with submicroscopic insertion of PML into RARA, leading to the expression of the PML-RARA transcripts as is evident by t(3;15) (q26;q13) cytogenetics and PML-RARA fusion by molecular assay. Fluorescent in situ hybridisation (FISH) or spectral karyotyping (SKy) will give the exact picture of chromosome rearrangements in complex karyotypes, which was not done in this present case as such facilities for those tests were not available at our centre. So it was a case of complex variant APML with t(3;15), indicating a novel breakpoint in chromosome 15q13 in spite of classic PML-RARA fusion. Our patient posed a therapeutic challenge as he had a structural congenital heart disease and use of anthracyclins in standard induction protocol for his intermediate risk category was a relative contraindication. Hence, he was started on alternate regimen of ATO with standard ATRA for induction and achieved successful remission.

## Conclusion

APML with complex or cryptic karyotype and those with variant RARA translocation, although rare, have to be considered when morphologically they resemble APML but lack classic t(15;17). Conventional cytogenetics and molecular studies are essential and relevant in all cases. Present case is a good example of this kind. Further, conventional cytogenetics reveals the variant category even though at molecular level it remains as classic APML. Also these translocations can exhibit significant differences in sensitivity to ATRA and ATO. Hence, unless proved in earlier studies to be ATRA or arsenic resistant, these patients have to be given the benefit of these targeted drugs as seen in our case.

## Figures and Tables

**Table 1.** Variant translocations and response to ATRA.

Translocation	Translocation partner	ATRA response
t(11;17) (q23;q21) [5]	PLZF (promyelocytic leukaemia zinc finger protein)	Resistant
t(5;17) (q35;q21) [6]	NPM (nucleophosmin)	Sensitive
t(11;17) (q13;q21) [7]	NUMA (nuclear mitotic apparatus)	Sensitive
der(17) [8]	STAT5b (signal transducer and activator of transcription)	Resistant
t(3;15) (q26;q13) (present case)		Sensitive
